# CRISPR/Cas9-mediated targeted mutation of the *E1* decreases photoperiod sensitivity, alters stem growth habits, and decreases branch number in soybean

**DOI:** 10.3389/fpls.2022.1066820

**Published:** 2022-12-14

**Authors:** Zhao Wan, Yingxiang Liu, Dandan Guo, Rong Fan, Yang Liu, Kun Xu, Jinlong Zhu, Le Quan, Wentian Lu, Xi Bai, Hong Zhai

**Affiliations:** ^1^ State Key Laboratory of Black Soils Conservation and Utilization, Northeast Institute of Geography and Agroecology, Chinese Academy of Sciences, Harbin, China; ^2^ College of Advanced Agricultural Sciences, University of Chinese Academy of Sciences, Beijing, China; ^3^ College of Life Science, Northeast Agricultural University, Harbin, China

**Keywords:** *E1*, CRISPR/Cas9, flowering time, photoperiod, soybean

## Abstract

The distribution of elite soybean (*Glycine max*) cultivars is limited due to their highly sensitive to photoperiod, which affects the flowering time and plant architecture. The recent emergence of CRISPR/Cas9 technology has uncovered new opportunities for genetic manipulation of soybean. The major maturity gene *E1* of soybean plays a critical role in soybean photoperiod response. Here, we performed CRISPR/Cas9-mediated targeted mutation of *E1* gene in soybean cultivar Tianlong1 carrying the dominant *E1* to investigate its precise function in photoperiod regulation, especially in plant architecture regulation. Four types of mutations in the *E1* coding region were generated. No off-target effects were observed, and homozygous trans-clean mutants without T-DNA were obtained. The photoperiod sensitivity of *e1* mutants decreased relative to the wild type plants; however, *e1* mutants still responded to photoperiod. Further analysis revealed that the homologs of *E1*, *E1*-*La*, and *E1*-*Lb*, were up-regulated in the *e1* mutants, indicating a genetic compensation response of *E1* and its homologs. The *e1* mutants exhibited significant changes in the architecture, including initiation of terminal flowering, formation of determinate stems, and decreased branch numbers. To identify *E1*-regulated genes related to plant architecture, transcriptome deep sequencing (RNA-seq) was used to compare the gene expression profiles in the stem tip of the wild-type soybean cultivar and the *e1* mutants. The expression of shoot identity gene *Dt1* was significantly decreased, while *Dt2* was significantly upregulated. Also, a set of MADS-box genes was up-regulated in the stem tip of *e1* mutants which might contribute to the determinate stem growth habit.

## 1 Introduction

Soybean (*Glycine max*) is highly sensitive to photoperiod; each soybean cultivar has to be planted in a narrow latitude range to obtain maximum yield, which limits the wide distribution of elite soybean varieties. Soybean has successfully adapted to a wide range of latitudes, attributed to natural variations in several major genes that control flowering and the presence of various allelic combinations of a series of major maturity loci. So far, 14 maturity loci have been identified in soybean, including *E1*-*E11*, *J*, *Tof5*, *Tof11*, and *Tof12* (Bernard, 1971; Buzzell, 1971; McBlain and Bernard, 1987; Ray et al., 1995; Bonato and Vello, 1999; Cober and Voldeng, 2001; Watanabe et al., 2009; Cober et al., 2010; Xia et al., 2012; Samanfar et al., 2017; Wang et al., 2019; Li et al., 2020; Lu et al., 2020).

Maturity locus *E1* was cloned using a map-based approach. It is assumed to be a legume-specific transcription factor with a putative nuclear localization signal and a region distantly related to the B3 domain (Xia et al., 2012). *E1* demonstrates a key role in repressing flowering and delaying maturity by repressing the expression of *GmMDEs, GmFT2a*, and *GmFT5a* in soybean. The *E1* locus is largely responsible for the variation in flowering time among soybean cultivars, and has the most prominent effect on flowering time and photoperiod sensitivity (Bernard, 1971; McBlain and Bernard, 1987; Upadhyay et al., 1994; Abe et al., 2003; Tsubokura et al., 2014). The expression level of the functional *E1* gene is strongly associated with the flowering time of soybean cultivars (Zhai et al., 2014b). *E3* and *E4* have been identified as homologues of the photoreceptor phytochrome A (Liu et al., 2008; Watanabe et al., 2009). The *E1* expression is modulated by *E3* and *E4* loci (Xia et al., 2012). The expansion of soybean cultivation in tropical regions can be attributed to the *J* locus, which controls the long-juvenile trait. *J* protein physically associates with the *E1* promoter to downregulate its transcription, relieving repression of *GmFT2a*/*5a* and promoting flowering under short days (Lu et al., 2017). *Time of Flowering 11* (*Tof11*) and *Time of Flowering 12* (*Tof12*) contributed to changes in flowering and early maturity in soybean crop evolution, demonstrating that their effects on flowering are genetically dependent on *E1* (Lu et al., 2020). *Time of Flowering 5* (*Tof5*), which promotes soybean flowering and adaptation to high latitudes, also acts downstream of *E1* (Dong et al., 2022). *E6* is a novel allele of *J* (Fang et al., 2021). *GmFT2a* was identified to be responsible for *E9* (Zhao et al., 2016). The FLOWERING LOCUS T orthologue, *GmFT4* is a strong candidate causal gene for maturity locus *E10*, and has been used in a breeding program (Samanfar et al., 2017). *GmFT4* functions as a flowering repressor, and is induced by *E1* (Zhai et al., 2014a). Another FT-like gene *GmFT1a* was proven to function as a flowering repressor, which is also induced by *E1* (Liu et al., 2018). Collectively, the *E1* gene serves as the hub of the photoperiodic responses of soybean, which is like a switch that controls control the photoperiod-dependent flowering.

Recent emergence of clustered regularly interspaced short palindromic repeats/CRISPR associated protein 9 (CRISPR/Cas9) technology has brought new opportunities to plant genetic engineering programs. CRISPR/Cas9 can be employed to make precise modification of genes controlling important agronomic traits (Shan et al., 2013; Xu et al., 2020). Cai et al. (2018) successfully edited *GmFT2a* and *GmFT5a* in soybean using the CRISPR/Cas9 system and generated late flowering mutants with high yield potential for the tropics (Cai et al., 2018). CRISPR/Cas9-mediated mutation of both *LUX1* and *LUX2* genes successfully obtained *lux1 lux2* double mutant, showing an extreme delay in flowering time and an insensitive response to day-length (Bu et al., 2021). Herein, we hypothesized that CRISPR/Cas9-mediated mutation of *E1* could create photoperiod insensitive germplasm, which can be used to expand the soybean genetic variations for breeding. CRISPR/Cas9-mediated mutation of *E1* has been conducted in soybean cultivar Jack (Han et al., 2019). However, CRISPR/Cas9 based genome editing technique relies on an efficient genetic transformation protocol, which is highly dependent on genotype. Currently, o nly a few soybean cultivars are amenable to genetic transformation (Donaldson and Simmonds, 2000). Jack is a model soybean cultivar for genetic transformation, but it carries a recessive *e1*-*as* allele, a leaky allele that retains partial *E1* function (Xia et al., 2012). The function of the *e1*-*as* allele is significantly weaker than the dominant *E1* allele. In this study, *Agrobacterium*-mediated transformation was used to introduce the CRISPR/Cas9 expression vector into soybean cultivar Tianlong1 carrying the dominant *E1* to analyze the precise effect of *E1* mutation, and to create novel germplasm in an elite background that can be used in soybean breeding. In addition, some new roles of *E1* were found, including the regulation of stem growth habit and branch number, the genetic compensation response of *E1* to its homolog *E1*-*La* and *E1*-*Lb*, and *E1*-regulated genes in stem tip. Our findings provides solid evidence that *E1* regulate s photoperiod by controlling the flowering time, stem growth habit and brunch number, and will inform future efforts in molecular breeding of photoperiod insensitive soybean cultivars.

## 2 Materials and methods

### 2.1 Plant materials and growth conditions

Soybean cultivar Tanlong1 carrying the dominant functional *E1* allele was used for genetic transformation. T_0_, T_1_ and T_2_ gene editing plants were planted in a growth chamber under long-day conditions (LD, 16 h/8 h, light/dark) and short-day conditions (SD, 12 h/12 h, light/dark) at 70% relative humidity and 200-300 μmol m^−2^ S^−1^ light fluency. Phenotypes of the T_2_ plants was recorded during the cultivation season (May to October) under a naturally LD conditions (LD, 16 h/8 h, light/dark) and artificially controlled short-day conditions (SD, 12 h/12 h, light/dark) in Harbin. The plant height and flowering time of the R1 stage (the first flower to appear) were recorded according to Fehr’s system (Fehr, 1971). Thirty plants of each genotype were measured and the data collected were subjected to statistical analysis.

### 2.2 Generation and identification of gene edited lines

CRISPR/Cas9 expression vector was constructed as described previously (Zhai et al., 2022). The CRISPR/Cas9 expression vector was transformed into *Agrobacterium* strain EHA105 by electroporation. Soybean transformation was performed using the cotyledonary node transformation system described previously (Flores et al., 2008). To identify the *e1* mutants, we extracted total genomic DNA from leaf samples of putative mutants using EasyPure^®^ Plant Genomic DNA Kit (TransGen, Beijing, China) according to the manufacturer’s instructions. Subsequently, PCR analysis was performed using *E1* sequence-specific primer sets (**Supplementary Table 1**). PCR products were detected using 1.5% agarose gel electrophoresis and then sequenced. The sequences of T_0_, T_1_, and T_2_ generation plants were analyzed using DSDecodeM to characterize the mutations induced by CRISPR/Cas9. Successfully edited lines were identified *via* sequence peaks and alignment to the reference sequences. The heterozygous mutants exhibited overlapping peaks near the target site, and the homozygous mutations showed single peaks at the target. The homozygous mutants were then identified by sequence alignment with the WT sequence. To screen and obtain *E1* targeted mutants without transgenic elements, we performed PCR analysis using *Bar* sequence-specific primer sets (**Supplementary Table 1**) to determine sgRNA/Cas9 on T-DNA elements. Potential off-target sites were predicted with the online tool: CRISPR-P (http://crispr.hzau.edu.cn/CRISPR2/) (Lei et al., 2014). Two potential off-target sites with the highest sequence identities to *E1* targets were analyzed. PCR was performed to amply the genome fragment containing the potential off-target sites, using primer pairs listed in **Supplementary Table 1**. PCR products were detected by 1.5% agarose gel electrophoresis and then sequenced.

### 2.3 Statistical analysis

Short-day promotion rate (SDHR) was calculated to determine the effect of photoperiod on flowering time, maturity and plant height as follows (Yan et al., 2009):


$$SDHR(\%)=\frac{V_{LD}-V_{SD}}{V_{LD}}\times 100\%$$

where *V_LD_* represents the phenotype value under LD, and *V_SD_* represents the phenotype value under SD.

### 2.4 RNA-seq assay

Wild-type soybean cultivar ‘Tianlong1’ and *e1* mutant were used for RNA-seq assay. Stem tips were collected 4 h after dawn from 28-day-old seedlings grown under LD conditions. Total RNA was extracted using TRIzol reagent (Invitrogen, Carlsbad, CA, USA) according to the manufacturer’s instructions. Sequencing cDNA libraries were generated according to the protocol of VAHTS ^®^ Universal V6 RNA-seq Library Prep Kit for Illumina (Vazyme, Nanjing, China). cDNA libraries were then constructed for sequencing on the Illumina NovaSeq 6000 S4 Seq system with 150-bp paired-end read lengths. Tophat2 was used to map clean reads to the soybean reference genome, Glycine max Wm82.a2.v1, from Phytozome (https://phytozome-next.jgi.doe.gov/). Differentially expressed genes (DEGs) were detected using DEGseq under the following parameters: fold change 2.00 and adjusted P-value (Q-value) 0.05. DEGs are listed in **Supplemental Data Set S1**. Sequence data of RNA-seq from this study were deposited in the National Genomics Data Center (SRA) database under accession number PRJCA010529.

### 2.5 Quantitative real-time PCR and semi-quantitative RT-PCR analyses

Total RNA was extracted from the leaves of 28-DAE-old plants of wild-type soybean TianLong1 and *E1* mutant grown under LD conditions (16 h/8 h, light/dark). Three independent replicates of the total RNA samples were prepared for each analysis.

Total RNA was extracted using TRIzol (Invitrogen, Carlsbad, CA, USA) method according to the manufacturer’s instructions. Subsequently, the RNA was treated with RNase-free recombinant DNase I (Takara, Dalian, China). The integrity of the RNA was determined using NanoDrop™ ND-2000c Spectrophotometer (Thermo Scientific, Wilmington, DE, USA). Equal amounts of isolated RNA were then reverse transcribed to cDNA using the SuperScript™ III Reverse Transcriptase kit. The quality of the cDNA samples was assessed by PCR using *GmTubulin A* (*TUA5*) specific primers.

Each cDNA sample was subjected to qRT-PCR analysis using the SYBR Green Master Mix (TransStart Top Green qPCR SuperMix, Beijing, China). The qRT-PCR analysis was performed on LightCycler^®^ 96 real-time PCR detection system (Roche, Switzerland) according to the manufacturer’s protocol. The measured Ct values were converted to relative copy-numbers using the 2^-ΔΔCt^ method. *TUA5* gene was used as an internal control to normalize all gene expression data. The qRT-PCR was performed using three fully independent biological replicates and each sample was run in triplicate. Raw data were standardized as described previously (Willems et al., 2008). Primers used for the qRT-PCR and semi-quantitative RT-PCR analyses are listed in **Supplementary Table 1**.

## 3 Results

### 3.1 CRISPR/Cas9-mediated mutations

In our previous study, to investigate the regulation of *GmMDE* genes by *E1*, we used CRISPR/Cas9-mediated mutation to inactivate *E1* in the soybean cultivar Tianlong1, which carries a functional *E1* allele (Zhai et al., 2022). In this study, we obtained four types of homozygous mutations. The *e1*-1 mutant line harbored a 244-bp deletion in the *E1* coding region, whereas the *e1*-2 mutant line harbored a 247-bp deletion in the *E1* coding region. The *e1*-3 mutant line harbored a 243-bp deletion in the *E1* coding region was obtained, whereas the *e1*-4 mutant line harbored a 209-bp deletion in the *E1* coding region. (**Supplemental Figure 1**). Notably, the *e1*-1 and *e1*-2 were frameshift mutations, causing premature termination of translation. The *e1*-1 and *e1*-2 produced a truncated protein encoding 98 and 97 amino acids, respectively, resulting in the deletion of all B3 domains and retaining part of the nuclear localization signal. The *e1*-3 and *e1*-4 produced a truncated protein encoding 153 and 165 amino acids, respectively, which retained part of the B3 domains and nuclear localization signal (**Supplementary Figure 2**). Potential off-target sites were predicted and the top 2 genomic regions of homology were selected as most likely off-target sites. Subsequently, each of these regions was amplified by PCR using genomic DNA from the mutant lines as template. The PCR products were further analyzed by Sanger Sequencing. Sequencing analysis did not reveal any potential off-target variants in the T_1_ mutants (**Supplementary Figure 3; Supplementary Table 2**). Thus CRISPR/Cas9 expression vector had specific edits at two targets. To obtain trans-clean mutants without T-DNA elements, we performed PCR to examine check whether there were traces of T-DNA in the mutants using *Bar* gene specific primers (**Supplementary Table 1**). Among the four T_1_ mutant lines, two were free of T-DNA in T_1_ generation derived from *e1*-1*, e1*-2, *e1*-3 and *e1*-4 (**Supplementary Table 3**). All the T_1_ mutant lines was flowering earlier than wild-type soybean cultivar Tianlong1 under LD conditions (**Supplementary Figure 4**).

### 3.2 Mutation of *E1* gene reduces photoperiod sensitivity in soybean

**Figure 1:**
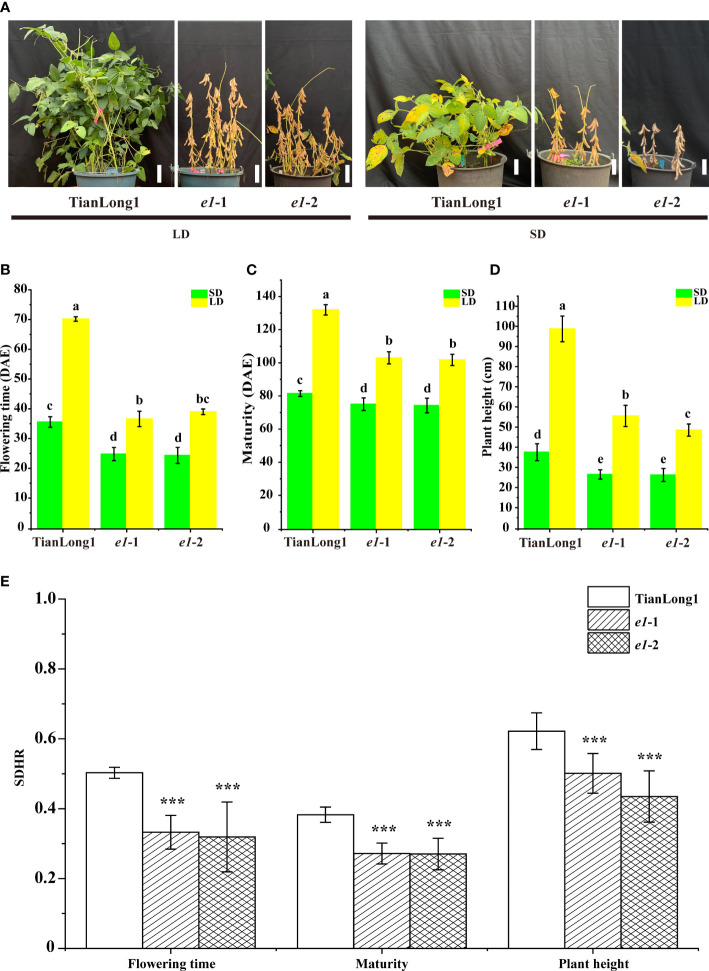
CRISPR/Cas9-induced *E1* mutant phenotypes under both LD and SD conditions. **(A)** Maturity of *E1* mutants and wild-type TianLong1 under LD and SD conditions. Scale bar, 10 cm. **(B)** Flowering time. **(C)** Time to maturity. **(D)** Plant height. **(E)** Short-day hastening rate. All data is shown as the mean values ± standard deviation (n = 20 plants). The diverse lowercase letter above the histogram bars in **(B–D)** by Two-Way ANOVA, suggests significant differences between the two panels (P > 0.05). The error bars in **(E)** indicates standard deviation. ***P<0.001, as determined by one-tailed Student’s *t-test*. DAE, day after emergence.

To analyze the effect of *E1* mutation on soybean photoperiod sensitivity, we planted T_2_ generation seeds of the homozygous mutant *e1*-1 and *e1*-2 without T-DNA elements under LD and SD conditions, respectively. The wild-type soybean cultivar Tianlong1 is extremely sensitive to photoperiod. The flowering time, maturation and plant height of Tianlong1 plants grown under LD conditions significantly differed from those under SD conditions (**Figures 1A-D**). Furthermore, the photoperiod sensitivity of the two *E1* mutants decreased greatly. Although the flowering time, maturation and plant height of two *E1* mutants under LD were different from those under SD conditions, differences in maturation and plant height between plants grown under LD and SD conditions decreased. The reproductive period R8 of the two *E1* mutants under SD conditions was about 25 days earlier than that under LD conditions, while the reproductive period R8 of Tianlong1 under SD conditions was more than 53 days earlier than that under LD conditions. The plant height of Tianlong1 under SD conditions was 60 cm shorter than that under LD conditions, while the plant height of the two *E1* mutants under SD conditions was about 25cm shorter than that under LD conditions. Collectively, these results indicate that loss of *E1* function reduces the photoperiod sensitivity of soybeans. Short-day hastening rate of flowering time, maturity, and plant height of the two *E1* mutants were significantly lower than that of wild-type Tianlong1 (**Figure 1E**).

### 3.3 The effect of *E1* mutation on *E1*-*Ls* and *GmFT2a*/*5a*

**Figure 2:**
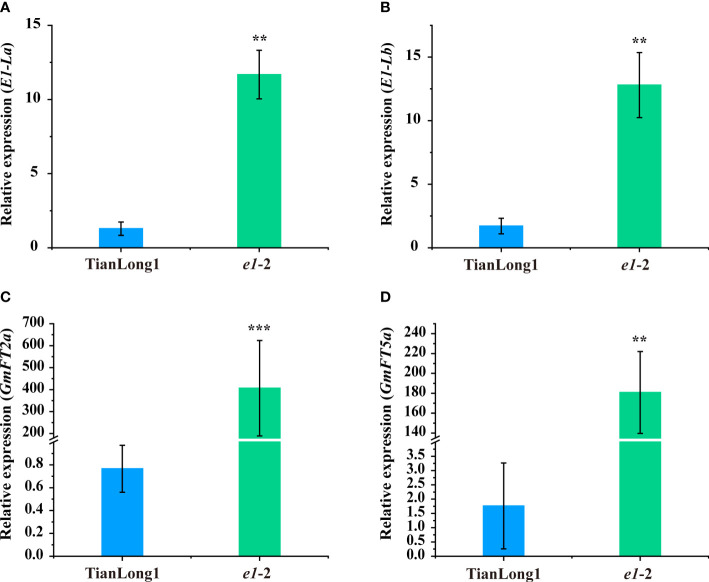
Expression analyses of *E1-Ls*/*GmFT2a*/*GmFT5a* in WT plants and mutants under LD conditions. **(A)** Expression analysis of *E1-La* under LD conditions. **(B)** Expression analysis of *E1-Lb* under LD conditions. **(C)** Expression analysis of *GmFT2a* under LD conditions. **(D)** Expression analysis of *GmFT5a* under LD conditions. Transcript levels were normalized to *TUA5*. Values represent the means of three biological replicates (n = 3 plants); error bars indicate standard deviation. **P<0.01; ***P<0.001, as determined by one-tailed Student’s *t-test*.

Since the two *E1* mutants were sensitive to photoperiod, we examined whether genetic compensation response of *E1* and its homologs exists. The soybean genome harbors two *E1* homologs, *E1-La* and *E1-Lb* (Xia et al., 2012). The expression of *E1-La* and *E1-Lb* in leaves of the *e1*-2 mutant and its wild-type soybean cultivar Tianlong1 were analyzed. In *e1*-2 mutant plants, the expression levels of two *E1-Ls* were significantly higher than those in the wild type (**Figures 2A, B**), indicating that a genetic compensation response of *E1* and its homologs exist.

In our previous study, *E1* overexpression repressed the expression of *GmFTs* (*GmFT2a* and *GmFT5a*), two homologs of *Arabidopsis FT* (Xia et al., 2012). To clarify the correlation between the expression of *E1* and *GmFT2a/5a* in flowering time regulation, we analyzed the transcript levels of *GmFT2a* and *GmFT5a* in the leaves of *e1*-2 mutant and wild-type (**Figures 2C, D**). *GmFT2a* and *GmFT5a* expression increased in *e1*-2 mutant compared to the wild-type Tianlong 1.

### 3.4 The *E1* mutants exhibit determinate stem growth and fewer branches

**Figure 3:**
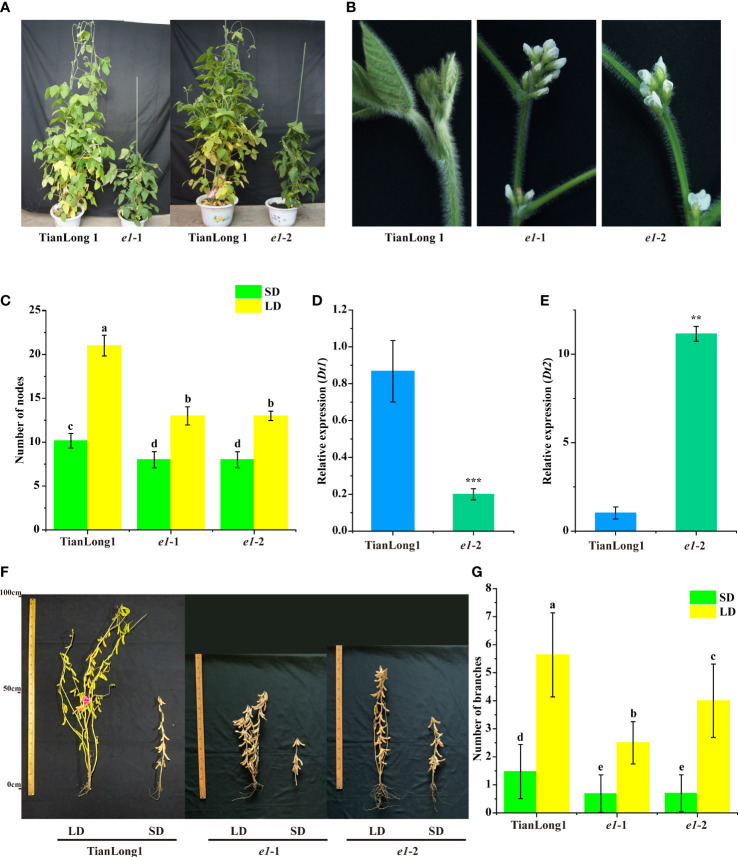
Loss of *E1* function changes the Stem Growth Habits of soybean. **(A)** Comparison of stem growth habit. **(B)** Morphology of the top of stems in determinate and indeterminate soybean. **(C)** Number of nodes. **(D)** Expression analysis of *Dt1* under LD conditions. **(E)** Expression analysis of *Dt2* under LD conditions. **(F)** Phenotypes of *E1* mutants and wild-type TianLong1 under LD and SD conditions. **(G)** Number of branches. Transcript levels were normalized to *TUA5*. Values represent the means of three biological replicates (n = 3 plants); error bars indicate standard deviation. **P<0.01; ***P<0.001, as determined by one-tailed Student’s *t-test*. All data is shown as the mean values ± standard deviation (n = 20 plants). The diverse lowercase letter above the histogram bars in (C and G) by Two-Way ANOVA, suggests significant differences between the two panels (P > 0.05).

The plant architecture of two *E1* mutants was investigated. Mutation of *E1* altered the stem growth habit of the mutants. The stem growth habit of the wild-type TianLong1 tended to be indeterminate under natural LD conditions and determinate under natural SD conditions (**Figure 3F**). Under a longer light period (16h:8 h light/dark), TianLong1 entirely exhibited indeterminate stem growth habit (**Figure 3A**). Notably, the two *E1* mutants exhibited determinate stem growth habits under both LD and SD conditions, characterized by early terminal flowering (**Figures 3B, F**), reduced plant height and decreased node number along the main stems (**Figures 1D**, **3C**).

Soybean stem growth habit is regulated by two major genes, *Dt1* and *Dt2*. *Dt1* specifies the indeterminate growth habit, which prevents terminal flowering, leading to taller plants (Liu et al., 2010; Tian et al., 2010; Liu et al., 2016). *Dt2* functions as a direct repressor of *Dt1*, promoting terminal flowering and leading to shorter plants (Liu et al., 2016). Here, we hypothesized that *E1* might regulate stem growth habit by modulating *Dt1* and *Dt2* genes. We thus measured the expression levels of *Dt1* and *Dt2* in the *e1*-2 mutant and wild-type Tianlong1 plants. *Dt1* expression was higher in the stem tips of wild type plants than in *e1*-2 mutant, while *Dt2* expression was significantly higher in the stem tips of *e1*-2 mutant than in wild-type stem tips (**Figures 3D, E**). These results indicate that *E1* regulates the stem growth habit *via* the *Dt2- Dt1* signaling pathway.

The branch number of wild type plants and two *E1* mutants were investigated. Mutation of *E1* altered the branch number. Wild type plants produced much more branches than the two *E1* mutants (**Figures 3F, G**). TianLong1 produced significantly more branch numbers under LD conditions than under SD conditions; however, the branch number of *E1* mutants was less different between SD and LD conditions (**Figures 3F, G**), indicating that mutation of *E1* reduces photoperiod sensitivity in branch number.

### 3.5 RNA-seq analysis of *E1* mutant

**Figure 4:**
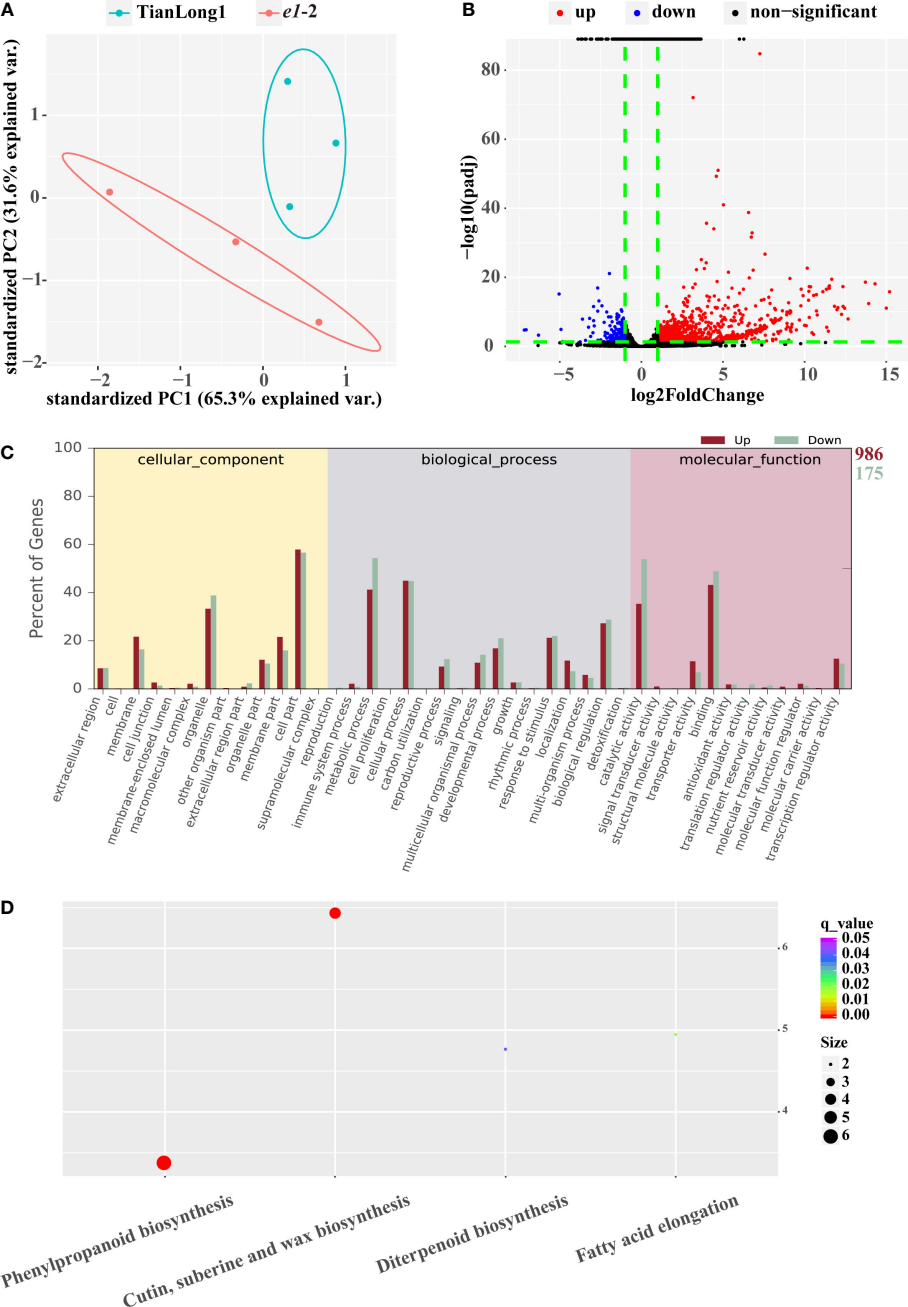
RNA Profiles in *e1*-2 and TianLong1. **(A)** PCA between *e1*-2 and TianLong1. **(B)** The volcano plot of mRNA expression signals in *e1*-2 and TianLong1. **(C, D)** GO (gene ontology) and KEGG (Kyoto encyclopedia of genes and genomes) enrichment analysis of total of 1161 DEGs.

Transcriptome sequencing (RNA-seq) and expression analysis of differentially expressed genes (DEGs) were performed further to understand the molecular differences between TianLong1 and *e1*-2. Similar gene expression levels were observed among three biological replicates (**Figure 4A**), WT and *e1*-2 were distinguished *via* principal component analysis (PCA). A total of 1161 DEGs were obtained by comparing the RNA-seq datasets (P< 0.05). Volcano plots were used to visualize the significant DEGs in *e1*-2 (**Figure 4B**). The red points in the graphic represent significantly upregulated genes, while the blue points in the graphic represent downregulated genes. Furthermore, we analyzed the gene ontology (GO) and Kyoto Encyclopedia of Genes and Genomes (KEGG) pathway enrichment of the differentially expressed mRNAs between *E1* mutants and controls. The most enriched terms GO terms included cell part (GO: cellular component), metabolic process (GO: biological process), binding (GO: molecular function), cellular process (GO: biological process), catalytic activity (GO: molecular function) and organelle (GO: cellular component) (**Figure 4C**). The most significantly enriched KEGG pathway was phenylpropanoid biosynthesis, Cutin, suberin and wax biosynthesis (**Figure 4D**).

**Figure 5:**
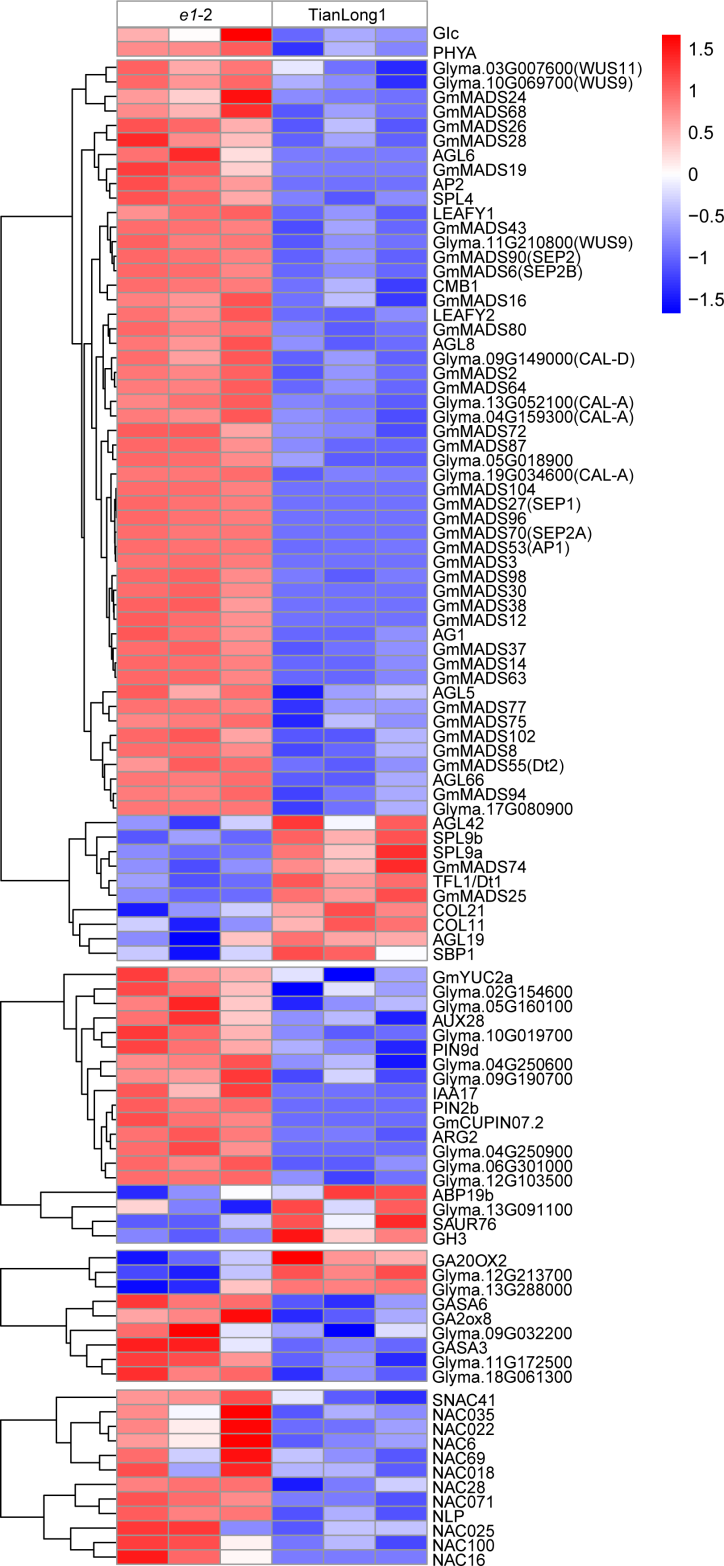
Heatmap showing the hierarchical clustering of DEGs in *e1*-2 and TianLong1.

The expressions of several flowering related genes were altered in the *E1* knockdown lines, consistent with the *e1*-2 early flowering phenotype, with most of the genes up-regulated. Among the 32 MADS-box genes, 28 were significantly up-regulated and 4 were down-regulated. Specifically, *E1* knockdown significantly up-regulated floral meristem identity genes, *LEAFY* (*LFY*) and *APETALA1* (*AP1*), and most floral organ identity genes. Furthermore, the expression of *SEPALLATA*(*SEP*), *CAULIFLOWER*(*CAL*) and *WUSCHEL*(*WUS*) genes were up-regulated. *E1* knockdown also affected the expression of multiple key factors in auxin and gibberellin signaling pathways. In particular, the expression of *PIN*, encoding auxin efflux carrier protein and *GA2OX*, the key enzymes in Gibberellin (GA) synthesis were upregulated. Also, 12 differentially expressed NAC transcription factors that mediate SAM formation were up-regulated (**Figure 5** and **Supplementary Table 4**).

## 4 Discussion

Studies have shown that *E1* confers the most prominent effect on photoperiod sensitivity in plants (Bernard, 1971; Cober et al., 1996; Watanabe et al., 2012; Xia et al., 2012). In soybean, genes that contribute to photoperiodic flowering and domestication, such as *E3*, *E4, J*, *Tof5 Tof11*, *Tof12, LUX1*, *LUX2*, and *Tof16* all function by modulating *E1* expression (Xia et al., 2012; Xu et al., 2015; Lu et al., 2017; Li et al., 2020; Lu et al., 2020; Bu et al., 2021; Dong et al., 2021), indicating that *E1* is the core regulator of soybean photoperiodic responses. *E1* regulates the photoperiod of soybean like a switch. CRISPR/Cas9 system has recently emerged as an effective method for targeted genome editing and gene function research. Here, we mutated the *E1* gene in soybean cultivar Tianlong1 carrying the dominant *E1 via* CRISPR/Cas9 system to study its function on the photoperiod response of soybean. Short-day hastening rate of flowering time, maturity, and plant height of the two *E1* mutants indicated that mutation of the *E1* gene reduces photoperiod sensitivity in soybean (**Figure 1E**).

However, the two *E1* mutants were still sensitive to photoperiod. Therefore, we examined whether the genetic compensation response of *E1* and its homologs exists. The results showed that *E1-Ls* were significantly up-regulated in *e1*-2 mutant plants relative to the wild type plants (**Figures 2A, B**), suggesting the existence of a genetic compensation response of *E1* and its homologs. These results also indicate that simultaneous mutation of *E1* and two homologs, *E1*-*la* and *E1*-*lb* might reduce photoperiod sensitivity more effectively.

In this study, the *e1* mutant bloomed and matured earlier than the wild type under both LD and SD conditions (**Figures 1B, C**). Previously, CRISPR/Cas9 was used to mutate *E1* in soybean cultivar Jack carrying the recessive *e1-as* allele to generate *e1* mutant (Han et al., 2019). The flowering time of mutants was significantly earlier than that of wild type plants under LD conditions. However, under SD condition, no significant difference in flowering time was observed between the wild type plants and mutants. The *e1-as* allele is a leaky allele retaining partial *E1* function, which as a flowering suppressor is significantly weaker than the *E1* allele (Xia et al., 2012; Zhai et al., 2015). In most soybean cultivars, *E1* is highly induced under LD conditions and repressed under SD conditions (Xia et al., 2012; Zhai et al., 2022). The weak function and low expression level of *e1-as* allele reduce the effect of *E1* in soybean cultivar Jack under SD conditions; therefore, *E1* knockdown in the cultivar does not change its flowering time under SD conditions. Thus, soybean cultivars carrying functional dominant *E1* alleles should be used to determine the precise function of *E1*.

Soybean is a typical photoperiod-sensitive short-day flowering plant. Its flowering, maturity and plant architecture, including plant height, branch number, node numbers of main stem and pods per plant are mainly regulated by photoperiod (Yan et al., 2009). Studies on the role of *E1* have primarily focused on flowering time, maturity and plant height (Xia et al., 2012; Zhang et al., 2016; Han et al., 2019), however, the effect of *E1* on the other agronomic traits related to photoperiodic sensitivity remains poorly understood. In this study, mutation of *E1* caused phenotypic changes in stem growth habits. The *E1* mutants exhibited determinate stem growth habits under both LD and SD conditions (**Figures 3A, B, F**). Also, *E1* knockdown decreased *Dt1* expression and increased *Dt2* expression in stem tips relative to the wild-type, consistent with the phenotypic changes (**Figures 3D, E**). *Dt1* and *Dt2* are the main genes regulating soybean stem growth habits (Liu et al., 2010; Tian et al., 2010; Ping et al., 2014; Liu et al., 2016). Specifically, *Dt2* functions as a direct repressor of *Dt1*, promoting terminal flowering, thus producing shorter plants (Liu et al., 2016). *Dt2* encodes a dominant MADS domain factor belonging to the APETALA1/SQUAMOSA (AP1/SQUA) subfamily (Ping et al., 2014). Besides *Dt2*, a set of genes encoding MADS domain factor were up-regulated (**Figure 5**). These MADS domain factor potentially regulate the stem growth habit of soybean and might contribute to downregulation of *Dt1* in *e1*-2 mutant.

In our previous study, we mapped the major QTL for branch number to the proximate to the *E1* gene, inferring that *E1* gene or neighboring genetic factor significantly contributes to the branch number (Yang et al., 2017). Recently, it was proved that *Dt2* reduces the branch number in soybean by activating the transcription of the *GmAP1* gene family (Liang et al., 2022). In this study, the two *E1* mutants produced fewer branches compared with wild-type Tianlong1 under both SD and LD conditions (**Figure 3G**). Meanwhile, we found that the expression of *Dt2* and *AP1* were up-regulated in the *e1*-2 mutants (**Figures 3E** and **5**). This study provides solid evidence that *E1* regulates branch number. The branch number is an important agronomic trait related to photoperiodic sensitivity. The branch number of soybean is genotype dependent, with some cultivars showing more sensitivity to photoperiod than others (Yan et al., 2009). This can be attributed to the diverse genetic variation of *E1* among soybean cultivars (Xu et al., 2013; Zhai et al., 2015; Li et al., 2017).

Gibberellin promotes shoot branching or tillering in plants, whereas mutation of the gene encoding GA synthesis enzyme decreases branching or tillering (Lo et al., 2008; Ni et al., 2015; Wu et al., 2020; Huang et al., 2021). Physiological observations and molecular studies suggest crosstalk between the GA and auxin, as well as with auxin transport. Auxin transport is reduced in GA mutants (Willige et al., 2011). In this study, RNA-seq analysis indicated that key enzymes in GA synthesis and *PIN*, encoding auxin efflux carrier protein were upregulated in *e1* mutant (**Figure 5**). This suggests that *E1* potentially regulates the branching type by modulating the genes involved in GA synthesis and auxin transport. Notably, hormonal crosstalk of GA and auxin might contribute to the decreased branching phenotype of *e1* mutants.

## Data availability statement

The datasets presented in this study can be found in online repositories. The names of the repository/repositories and accession number(s) can be found in the article/**Supplementary Material**.

## Author contributions

HZ designed and supervised this research. ZW and YL conducted the experiments and analyzed the data. DG, RF, YL, LQ, and WL conducted the field trial. KX, JZ, and XB provided advice on experimental implementation. ZW and HZ prepared the manuscript. All authors contributed to the article and approved the submitted version.
