# Supplementary material for: CRISPR/Cas9-mediated targeted mutation of the E1 decreases photoperiod sensitivity, alters stem growth habits, and decreases branch number in soybean
Source: Front Plant Sci. 2022 Dec 14;13:1066820. doi: 10.3389/fpls.2022.1066820 (PMC9794841; doi:10.3389/fpls.2022.1066820)
Supplement: Supplementary file 1 [file Table_1.docx]

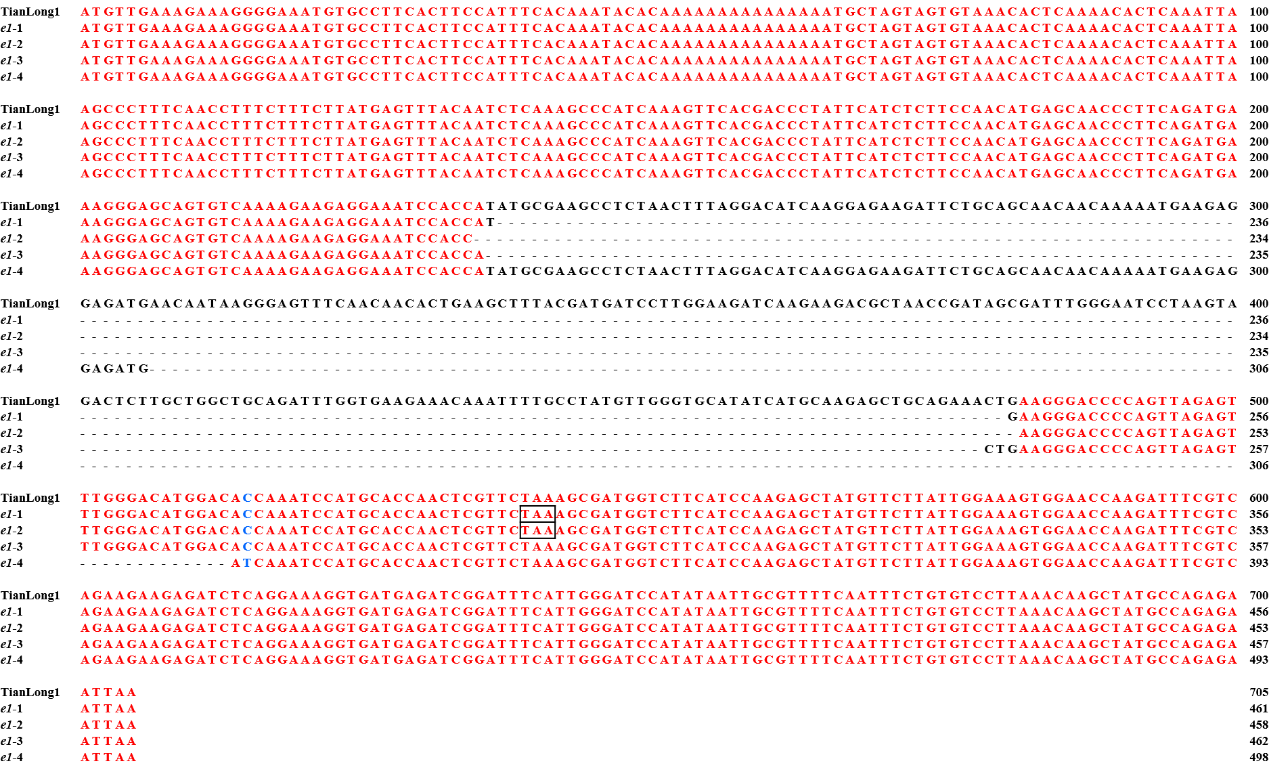


**Supplementary Figure 1.** Alignment of *E1* mutations nucleotide sequence with wild type. The premature translation stop codons for mutations were marked by black boxes.


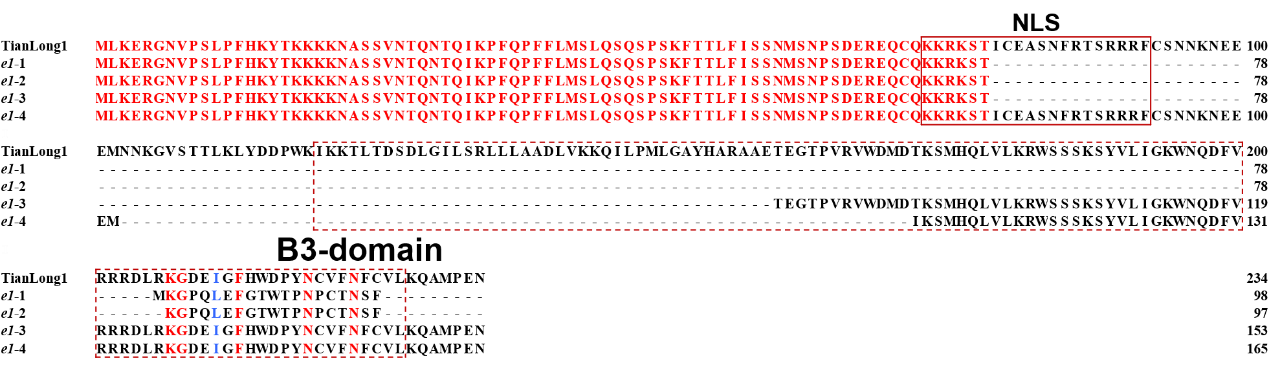


**Supplementary Figure 2.** Alignment of *E1* mutations amino acid sequence with wild type. Nuclear location signal marked by red squares. The B3-like domain was marked by dashed red squares. NLS, nuclear localization signal.

**
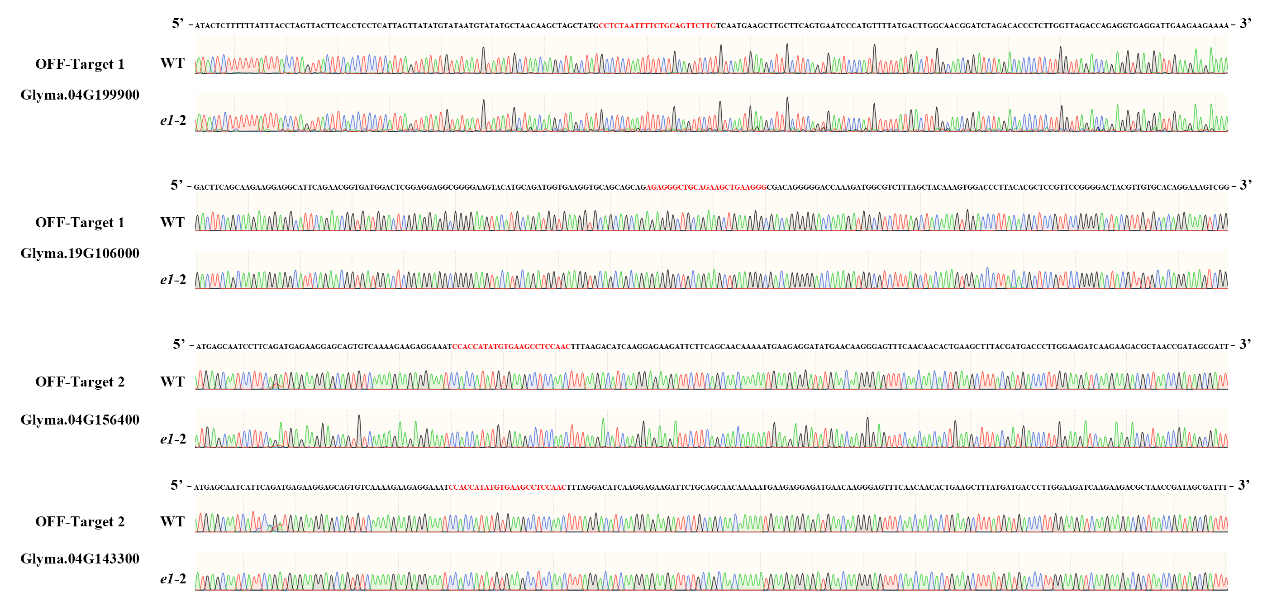
**

**Supplementary Figure 3.** Analysis of potential off-target sequences in T_1_ transgenic plants. The dotted line indicates that the potential off-target site exists in the antisense strand.

**
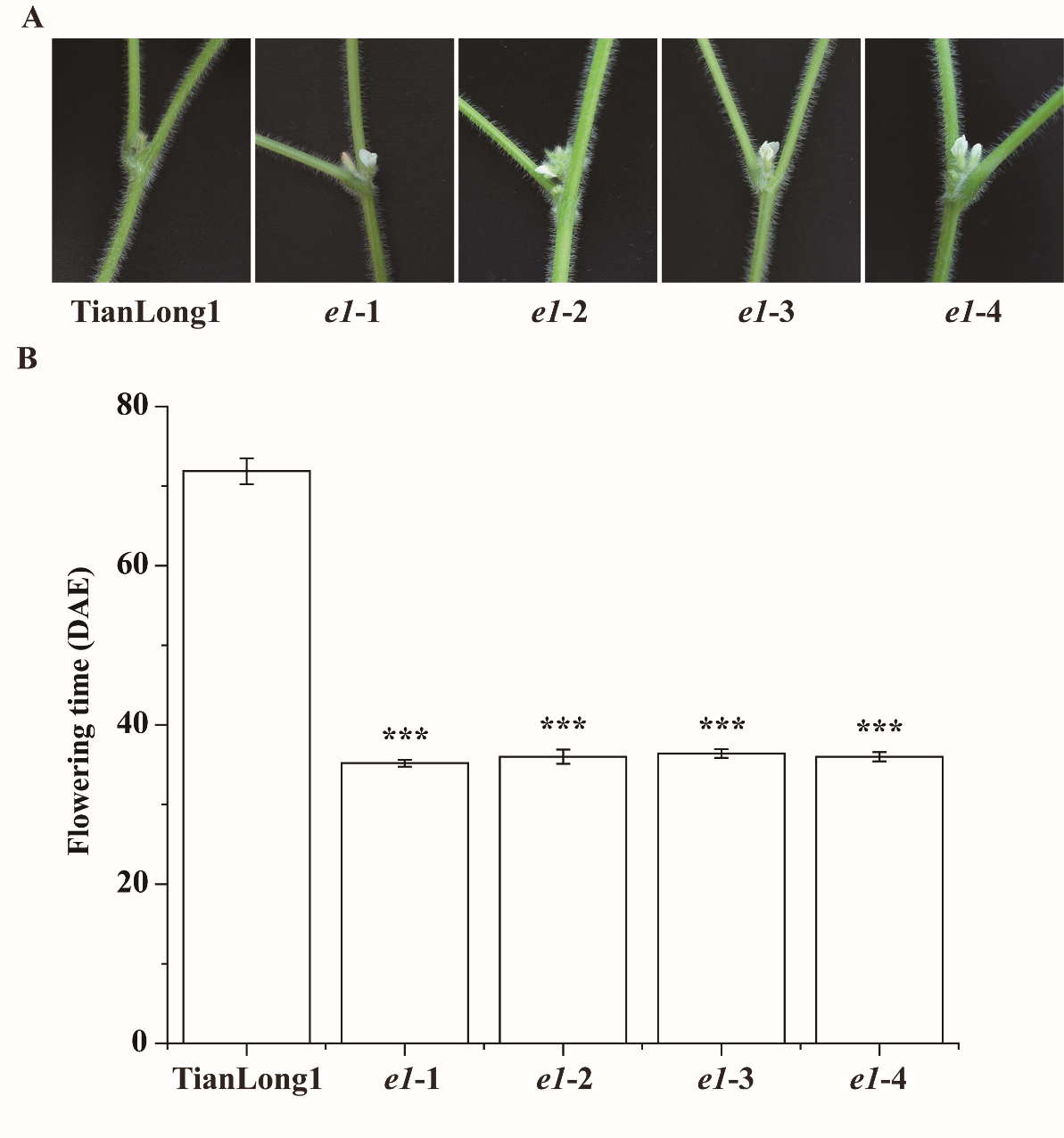
**

**Supplementary Figure 4.** Flowering time in *e1* mutant of T_1_ generation. (A) Compare with *e1* mutation and TianLong1 under LD conditions. (B) Flowering time. The error bars in (B) indicates standard deviation. ***P<0.001, as determined by one-tailed Student’s t-test.

**Supplementary Table 1** Primer sequences used in this study

| **Primer** | **Primer sequence (5’→3’)** | **Purpose** |
| --- | --- | --- |
| *E1-F* | AACACTCAAATTAAGCCCTTTCAACCT | To amplify the target site(*E1*) |
| *E1-R* | ACCAGGAAGTACTAGTGCAATGAGTC |  |
| *TUA5-F* | CCTTCTGTTGTTCCTGGTGGT | To amplify the reference gene (*GmTubulinA*) |
| *TUA5-R* | CAAACTTGTGGTCAATGCGAG |  |
| *Dt1-F* | GCTGGTTTTGCCACTGCAA | qRT-PCR experiment (*GmDT1*) |
| *Dt1-R* | AAGTTATTTGAAGCCACATGTGAA |  |
| *Dt2-F* | AGACAATGCAAGGAGGAACACCA | qRT-PCR experiment (*GmDT2*) |
| *Dt2-R* | CCTAACTAGTCAGACATGCAGCGC |  |
| *GmFT2a-F* | ATCCCGATGCACCTAGCCCA | qRT-PCR experiment (*GmFT2a*) |
| *GmFT2a-R* | ACACCAAACGATGAATCCCCA |  |
| *GmFT5a-F* | AGCCCGAACCCTTCAGTAGGGA | qRT-PCR experiment (*GmFT5a*) |
| *GmFT5a-R* | GGTGATGACAGTGTCTCTGCCCA |  |
| *E1La-F* | AAACACTCAAAGCCCGATCA | qRT-PCR experiment(*E1*-*La*) |
| *E1La-R* | ATCCTCTTCATTTTTGTTGCTGA |  |
| *E1Lb-F* | GTGTAAACACTCAAAGTCCTT | qRT-PCR experiment(*E1*-*Lb*) |
| *E1Lb-R* | CTCCTCTTCATTTTTGTTGCTGC |  |
| *e1-sg1-1-F* | TGCAAACACTCAAAGCCCGAT | To amplify the potential off target site 1-1 |
| *e1-sg1-1-R* | ATAGGCATAATATATTGGCCATTGC |  |
| *e1-sg1-2-F* | GTGTAAACACTCAAAGTCCTT | To amplify the potential off target site 1-2 |
| *e1-sg1-2-R* | GATTTGAAAGTAAAATAAAGCTAACACTT |  |
| *e1-sg2-1-F* | AGGCCCTTATGTTGGACGAA | To amplify the potential off target site 2-1 |
| *e1-sg2-1-R* | TCAAATGAGGCAGGGTTTGAA |  |
| *e1-sg2-2-F* | CTCACCTCCTCCCCAAGAAC | To amplify the potential off target site 2-2 |
| *e1-sg2-2-R* | CTGGTGGACAGGTAGCTTCG |  |
| *Bar-F* | TGCCAGTTCCCGTGCTTGAA | To amplify a part of T-DNA elements (*Bar*) |
| *Bar-R* | CTGCACCATCGTCAACCACTA |  |

**Supplementary Table 2** Analysis of potential off-target sites in T_1_ generation

| **Target** | **Physcial position** | **Position** | **Target sequence** | **No. of mismatch** | **No. of plants sequenced** | **No. of off-target plants** |
| --- | --- | --- | --- | --- | --- | --- |
| Target1:CAAGAGCTGCAGAAACTGAAGGG | Glyma.04G199900 4: -44066653 | Intron | CAAGAaCTGCAGAAAaTtAgAGG | 4 | 22 | 0 |
|  | Glyma.19G106000 19: +35482272 | Exon | agAgGGCTGCAGAAgCTGAAGGG | 4 | 22 | 0 |
| Target2:GTTAGAGGCTTCGCATATGGTGG | Glyma.04G156400 4: -28294190 | Exon | GTTgGAGGCTTCaCATATGGTGG | 2 | 22 | 0 |
|  | Glyma.04G143300 18: -25740216 | Exon | GTTgGAGGCTTCaCATATGGTGG | 2 | 22 | 0 |

The red letters represent mismatched bases. +, sense strand. -, antisense strand.

**Supplementary Table 3** Transgene-clean homozygous mutants from T_1_ generation

| T_1_ Generation | No. of plants identified | No. of trans-clean plants |
| --- | --- | --- |
| *e1*-1 | 6 | 2 |
| *e1*-2 | 5 | 4 |
| *e1*-3 | 1 | 0 |
| *e1*-4 | 9 | 0 |
